# Mandibular Ramus Fractures: A Case Series of Diversity in Rarity

**DOI:** 10.7759/cureus.30471

**Published:** 2022-10-19

**Authors:** Shreya S Pawar, Nitin D Bhola, Anchal Agarwal

**Affiliations:** 1 Oral and Maxillofacial Surgery, Sharad Pawar Dental College and Hospital, Datta Meghe Institute of Medical Sciences, Wardha, IND

**Keywords:** mandibular fracture, plating, open reduction internal fixation, classification, ramus fractures

## Abstract

Mandibular ramus fracture is usually minimally displaced as it is surrounded by the medial pterygoid medially, masseter laterally, and the pterygomasseteric sling inferiorly. They are commonly caused either by road traffic accidents or interpersonal violence. Ramus fracture is usually seen in conjunction with other mandibular fractures and is seldom found alone. The ramus is located at the congregation of the dentate and the non-dentate parts of the mandible. Ramus fractures are generally managed by closed reduction when minimally displaced but this technique has its disadvantages like poor maintenance of oral hygiene and prolonged healing time. It can get fractured in various patterns. Owing to the presence of anatomical structures on either side of the ramus and the orientation of the fracture line, the treatment plan varies in each case to prevent paresthesia by preserving the inferior alveolar nerve. This article has demonstrated four distinct kinds of mandibular ramus fractures and their management with open reduction internal fixation (ORIF).

## Introduction

The human face is subdivided into the upper, mid, and lower face which comprises the mandible. The incidence of mandibular fractures in India is 57% out of all maxillofacial fractures [1]. Coronoid and ramus fractures are the least prevalent among the mandible subsites.

The mandibular ramus is located at the congregation of dentate and non-dentate parts of the mandible. The ramus of the mandible is fractured mainly due to road traffic accidents, falls, and assaults. They are common in men in the age group of 25-34 years [2]. Ramal fractures are the second least commonly fractured subsite of the mandible after the coronoid fracture [3]. The incidence of ramus fracture is 3.09% of all mandibular fractures [4]. A fractured ramus clinically presents with pain and swelling along with trismus. There could be associated anterior open bite or occlusal discrepancies. 

Management of the mandibular fracture aims to re-establish esthetics and function by restoring pre-trauma dental occlusion and standard mouth opening while also reducing the displaced fracture. The use of open reduction internal fixation (ORIF) in the treatment of mandible fractures has revolutionised the field, reducing the need for rigid maxillomandibular fixation (MMF) after surgery [5]. We present to you a case series of four types of ramus fracture that were presented to our institute at Acharya Vinoba Bhave Rural Hospital, Sawangi, Wardha at the “Inpatient Department of Oral and Maxillofacial Surgery” along with their management.

## Case presentation

Case I

**Figure 1:**
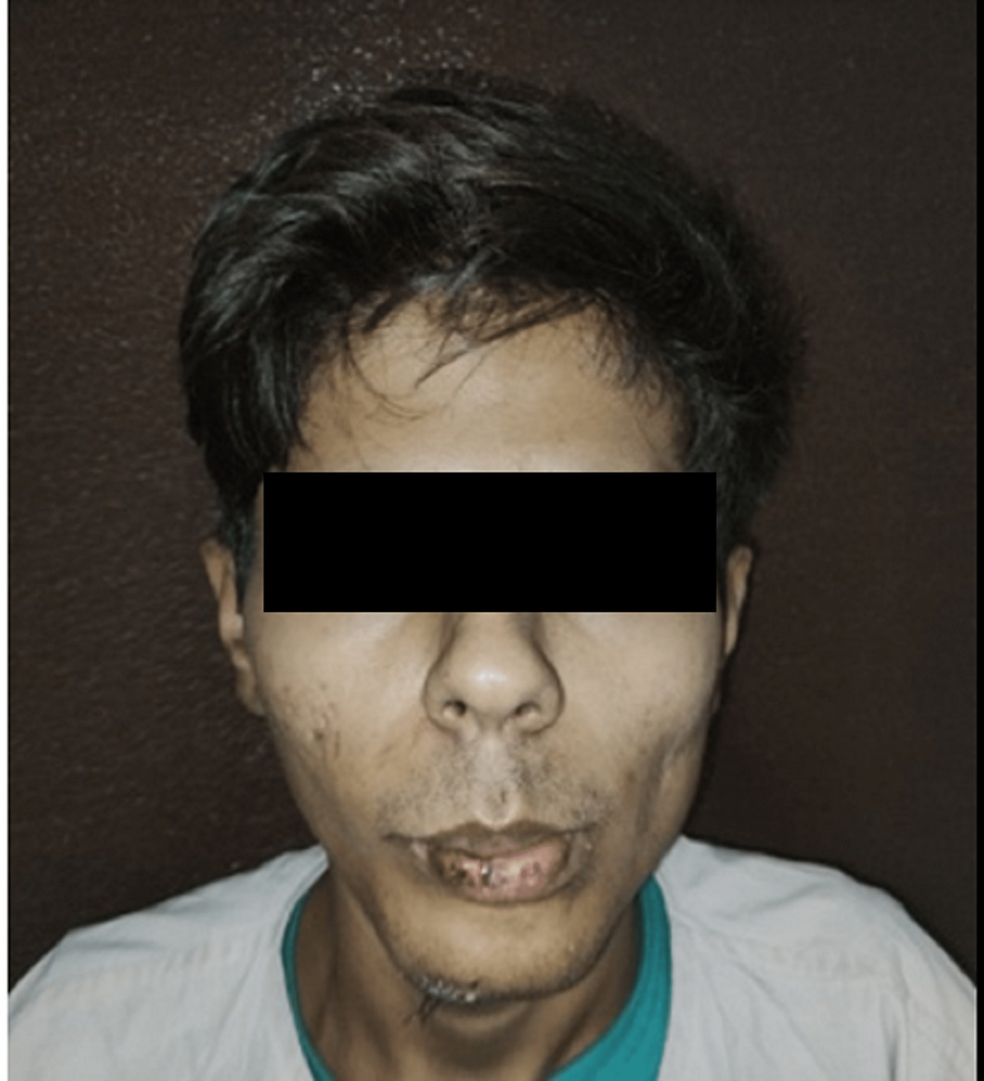
Pre-operative frontal view of case I

**Figure 2:**
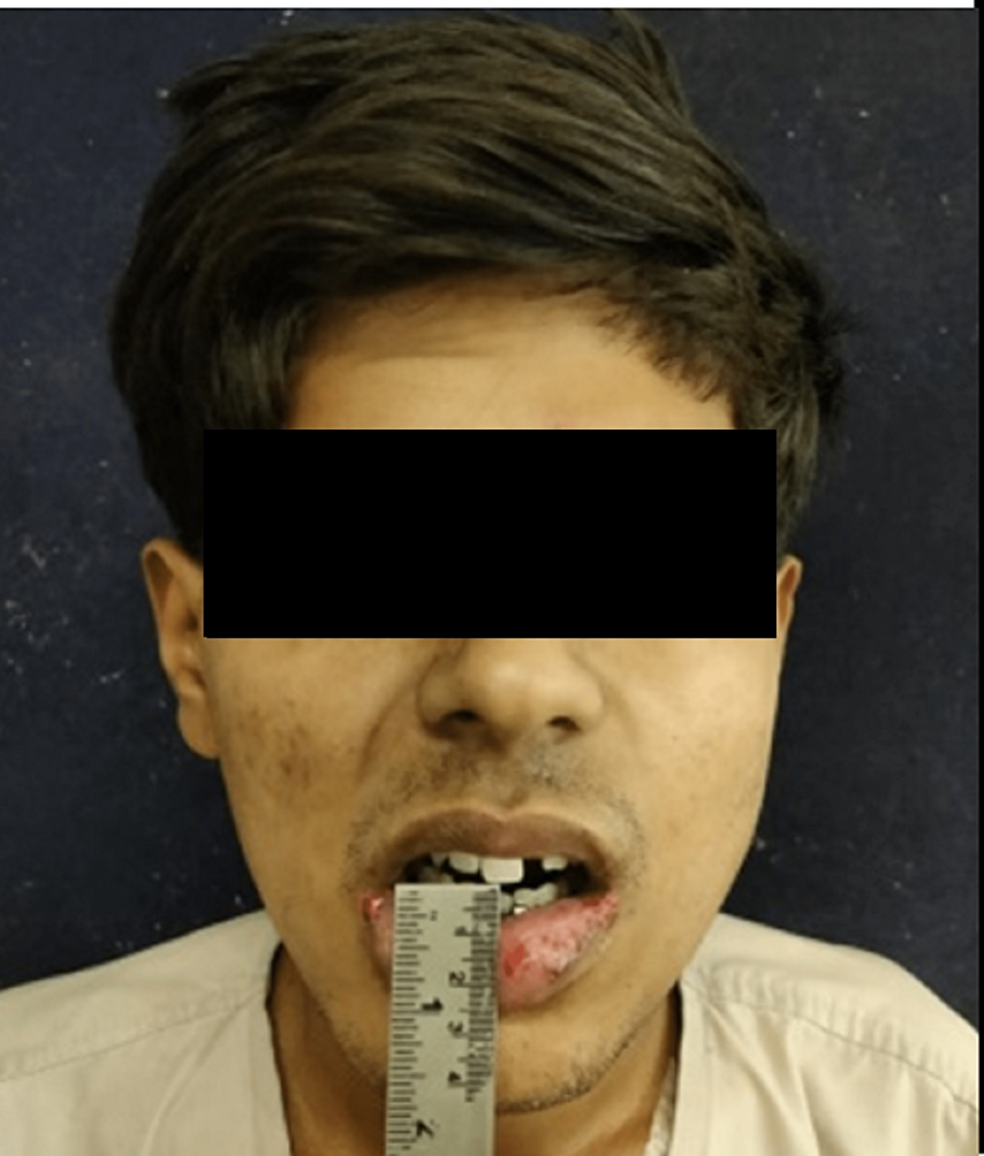
Pre-operative mouth opening of case I Mouth opening - 3mm

A 23-year-old man was presented to the casualty soon after he went through a road traffic accident (RTA) due to a slip on his bike. The patient reported with sutured contused lacerated wound over the chin and lower lip (Figures 1, 2). On taking a detailed history, it was found that the patient had retrograde amnesia along with an episode of vomiting.

**Figure 3:**
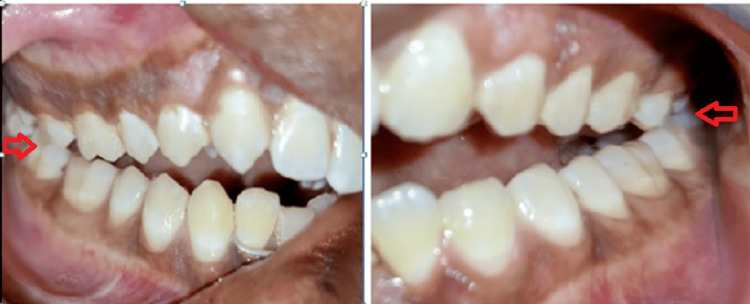
Pre-operative occlusion Bilateral posterior molar gagging causes anterior open bite. Left-hand side (LHS) panel - The arrow shows molar gagging of the right side; Right-hand side (RHS) panel - The arrow shows molar gagging of the left side

**Figure 4:**
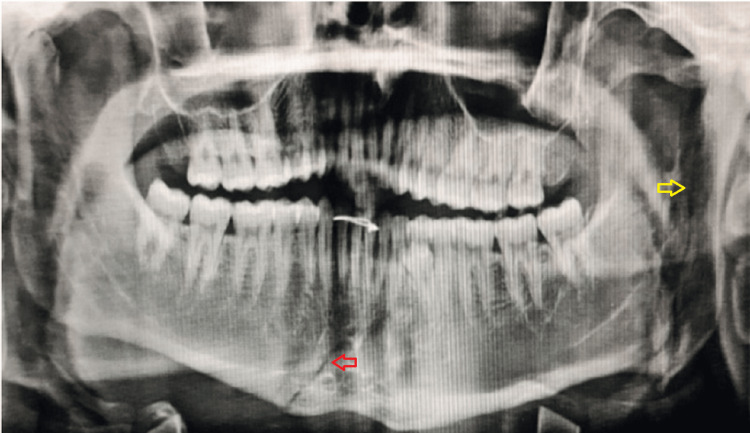
Pre-operative OPG of case I OPG: Orthopantomogram The yellow arrow shows the vertical fracture line from the sigmoid notch to the inferior border of the mandible on the left side. The red arrow shows the fracture line extending from 32 to the inferior border of the mandible corresponding to the mandibular right premolar region.

**Figure 5:**
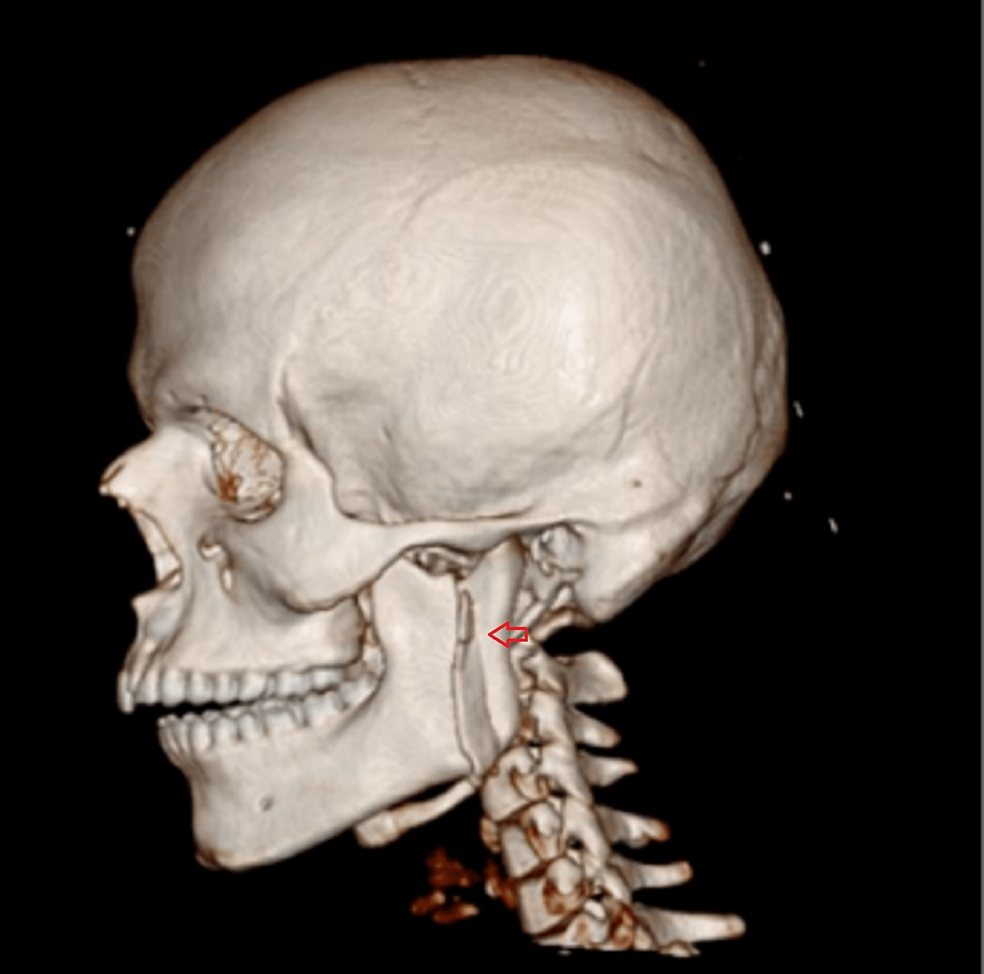
3D reconstructed computed tomographic scan of case I The image shows the left ramus fracture- fracture line running from the mandibular notch to the lower boundary of the mandible (red arrow).

On further examination, it was noted that the patient had step and tenderness over the left ramus and right parasymphysis region and tenderness over the right preauricular region. The patient had reduced mouth opening with restricted jaw movements and premature gagging of the molars bilaterally causing an anterior open bite (Figure 3). He had intersegmental mobility between the lower right central and lateral incisors which was reduced with a Briddle’s wire in the casualty. A computed tomographic scan with the 3D reconstruction of the head (Figures 4, 5) was done for the patient which revealed a fracture line that travelled from the top of the subsigmoid notch traversing through the ramus to the lower boundary of the fracture line. The patient was diagnosed with a left ramus fracture in conjunction with right parasymphysis and high condylar fracture through a thorough clinical and radiographic examination.

**Figure 6:**
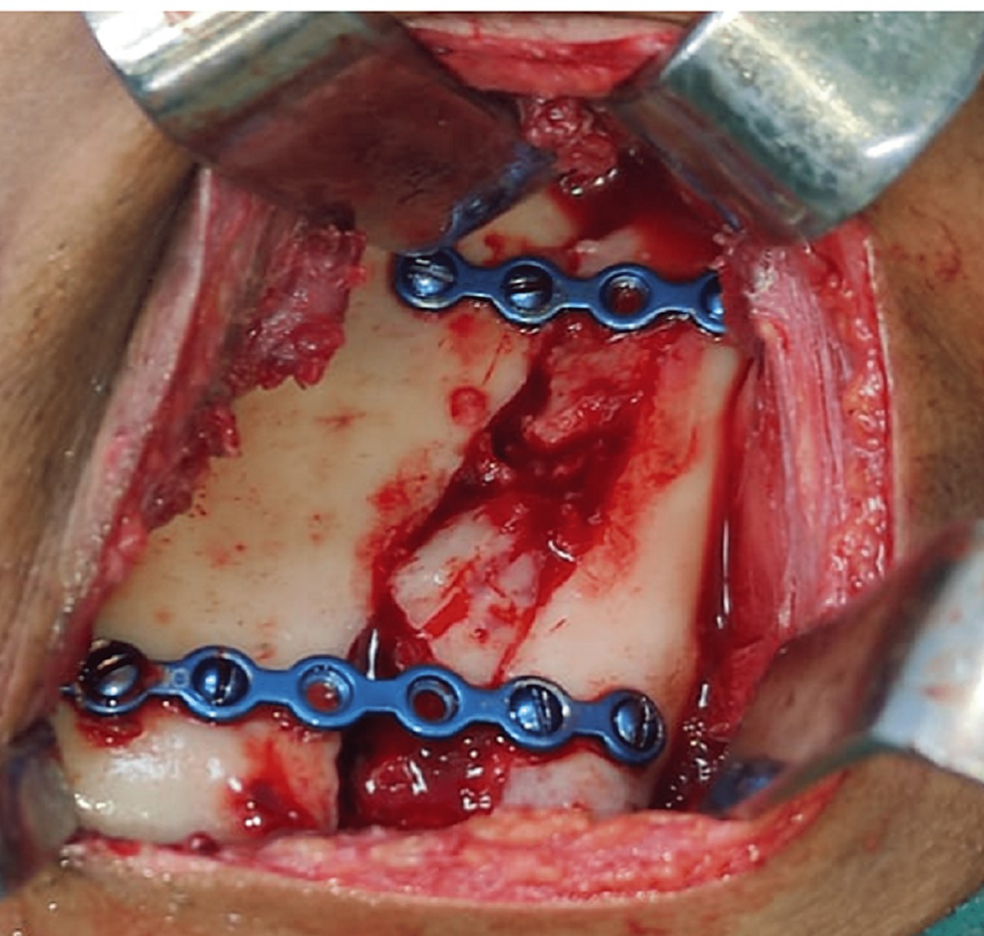
Open reduction and internal fixation of the left ramus fracture

**Figure 7:**
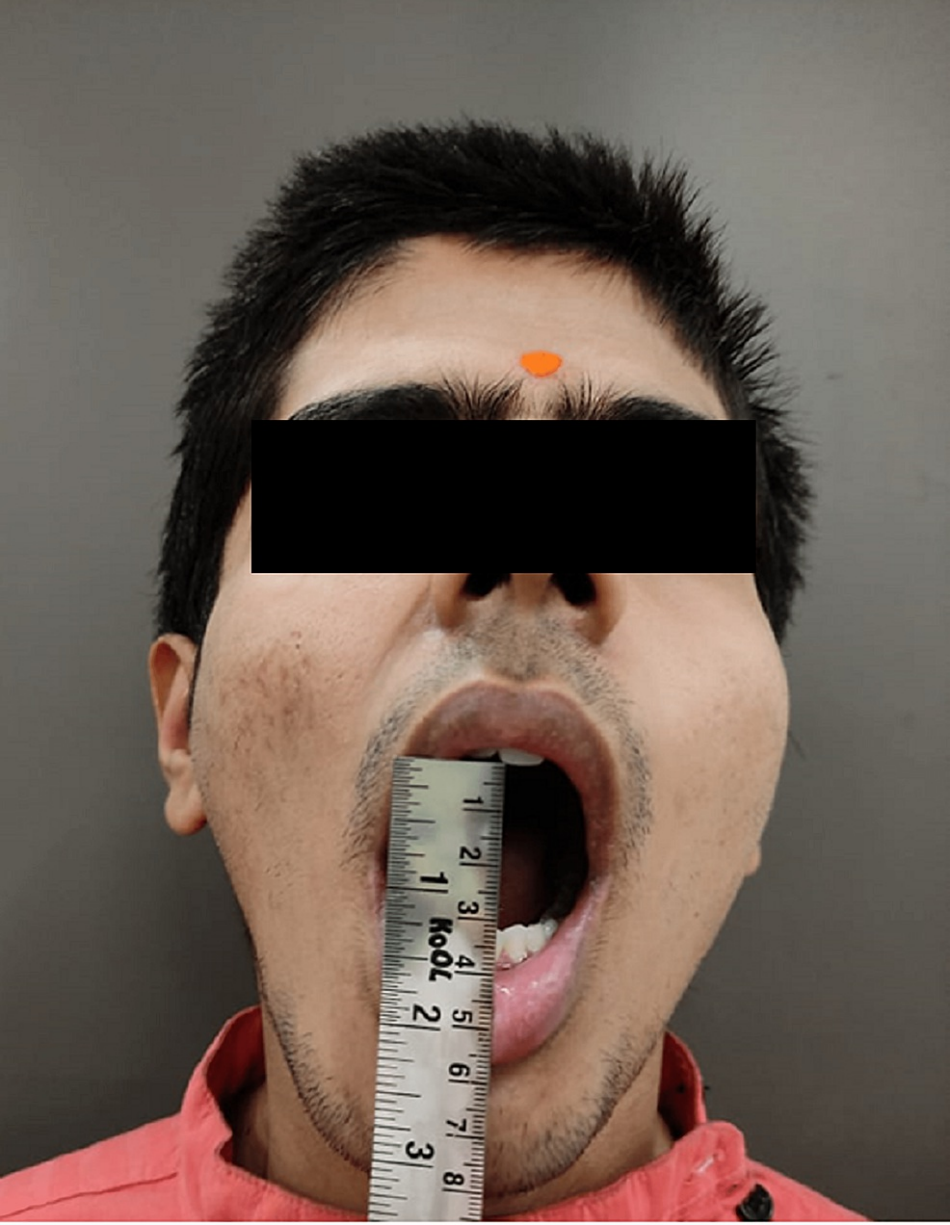
Post-operative mouth opening of case I Mouth opening - 33mm

**Figure 8:**
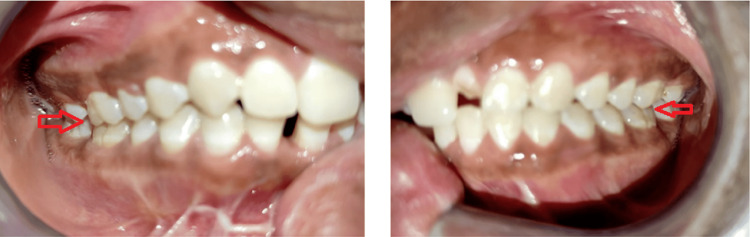
Post-operative occlusion Occlusion - Bilaterally Stable The arrow in the left-hand side (LHS) panel shows a stable occlusion over the right side whereas the arrow in the right-hand side (RHS) panel shows a stable occlusion over the left side

**Figure 9:**
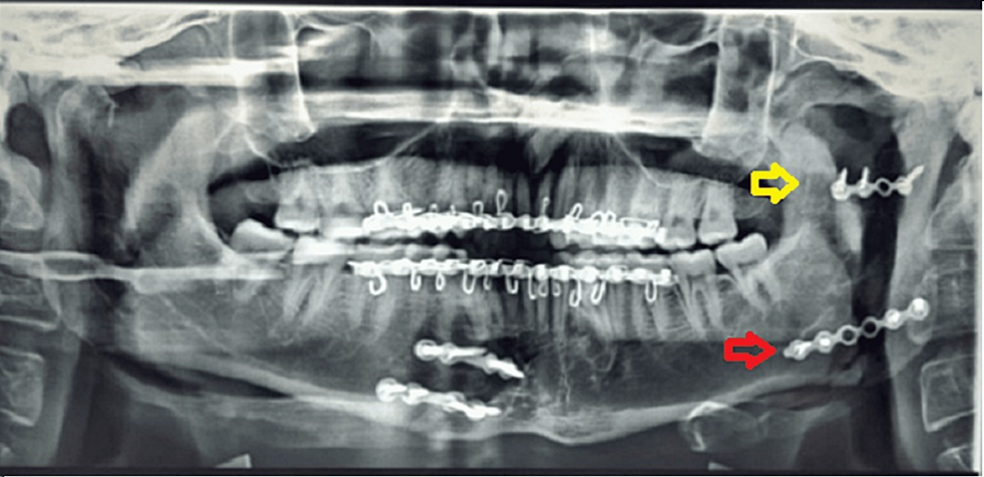
Post-operative OPG OPG: Orthopantomogram The image shows a mini plate over the upper border of the fracture line above the lingula (yellow arrow) and others at the lower border of the mandible (red arrow).

After taking formal consent, the patient was managed with open reduction and internal fixation (ORIF) under general anaesthesia of the fractured left ramus through periangular incision whereas the parasymphysis was reduced using a mandibular vestibular incision. The fixation of the ramus was done with a 2mm 4-hole continuous non-compression mini-plate just below the subsigmoid notch and above the inferior alveolar nerve canal and a 2mm 6-hole continuous non-compression mini-plate at the lower border of the mandible (Figure 6). The patient was on observation for five days. The surgical site showed healing and the occlusion was stable bilaterally. The post-operative orthopantomogram showed a successful reduction of the fracture site (Figures 7-9).

Case II

**Figure 10:**
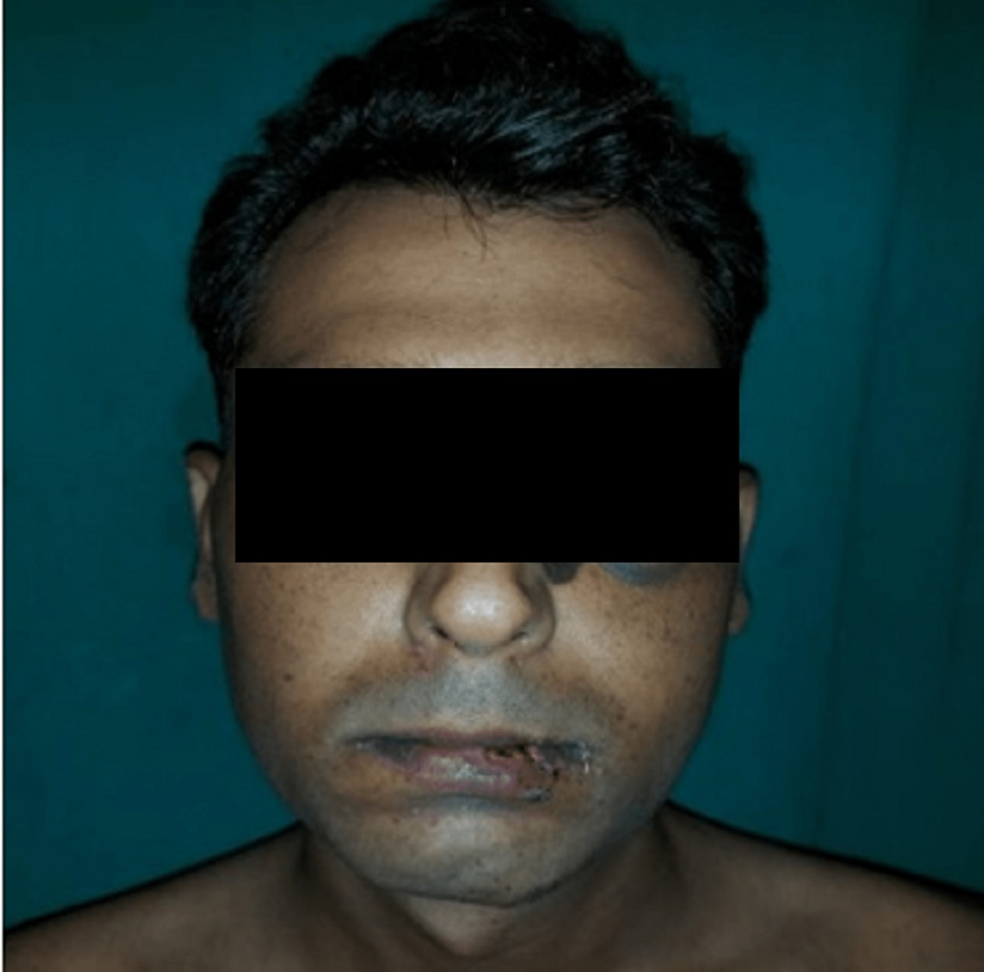
Pre-operative frontal view of case II

**Figure 11:**
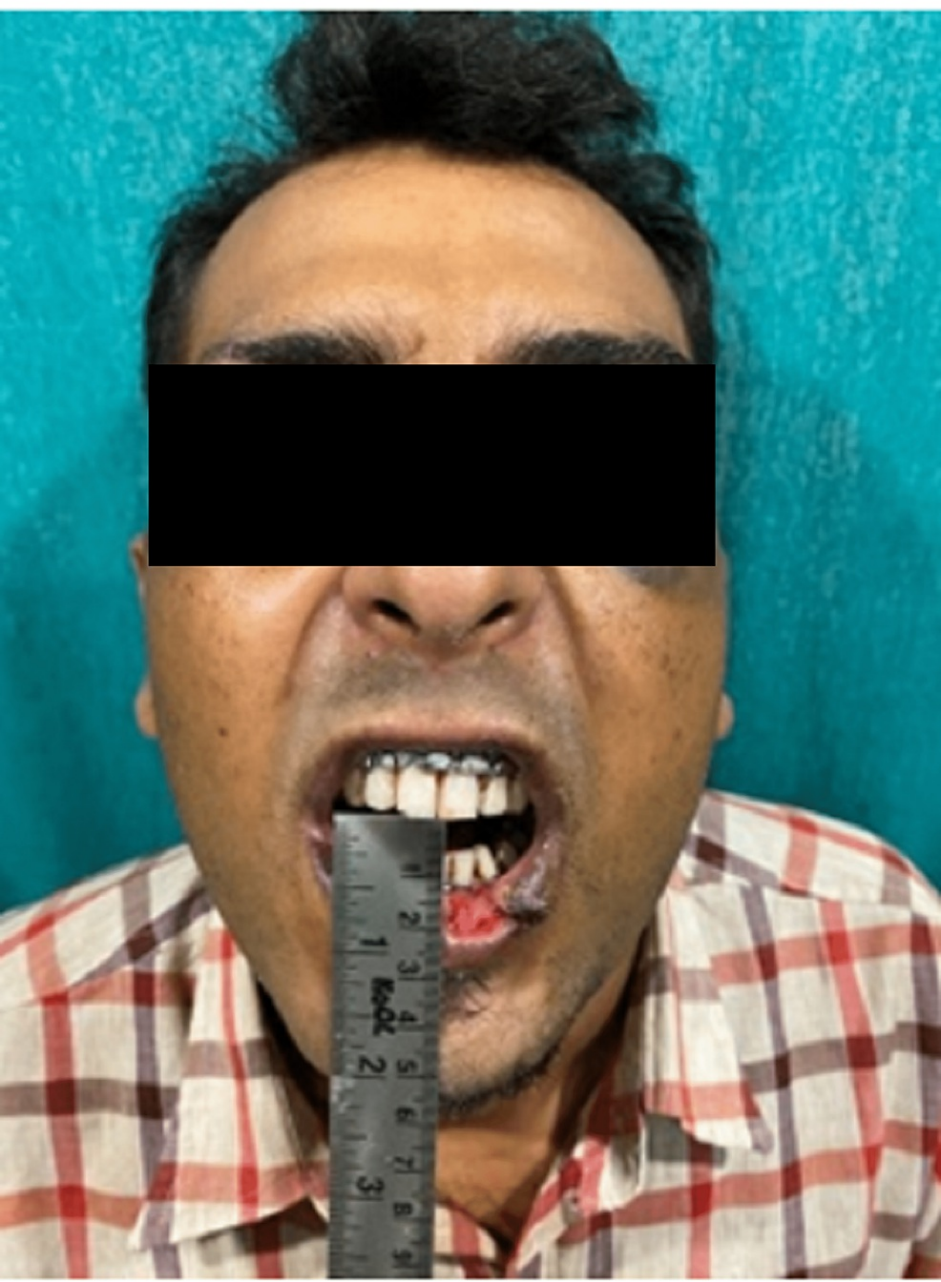
Pre-operative mouth opening of case II Mouth opening - 8mm (reduced)

This 30-year-old male was brought to the casualty after meeting a road traffic accident due to a bike slip. The patient had presented with a step over the right parasymphysis region (Figures 10, 11). He had tenderness over the left ramus region and had a bilateral open bite with a mouth opening of 6mm.

**Figure 12:**
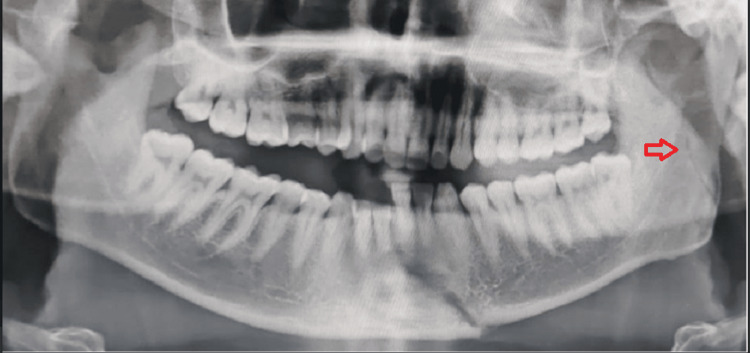
Pre-operative OPG OPG: Orthopantomogram The image shows a Type II ramus fracture with a fracture line extending obliquely from the coronoid to the posterior border of the mandible (red arrow).

**Figure 13:**
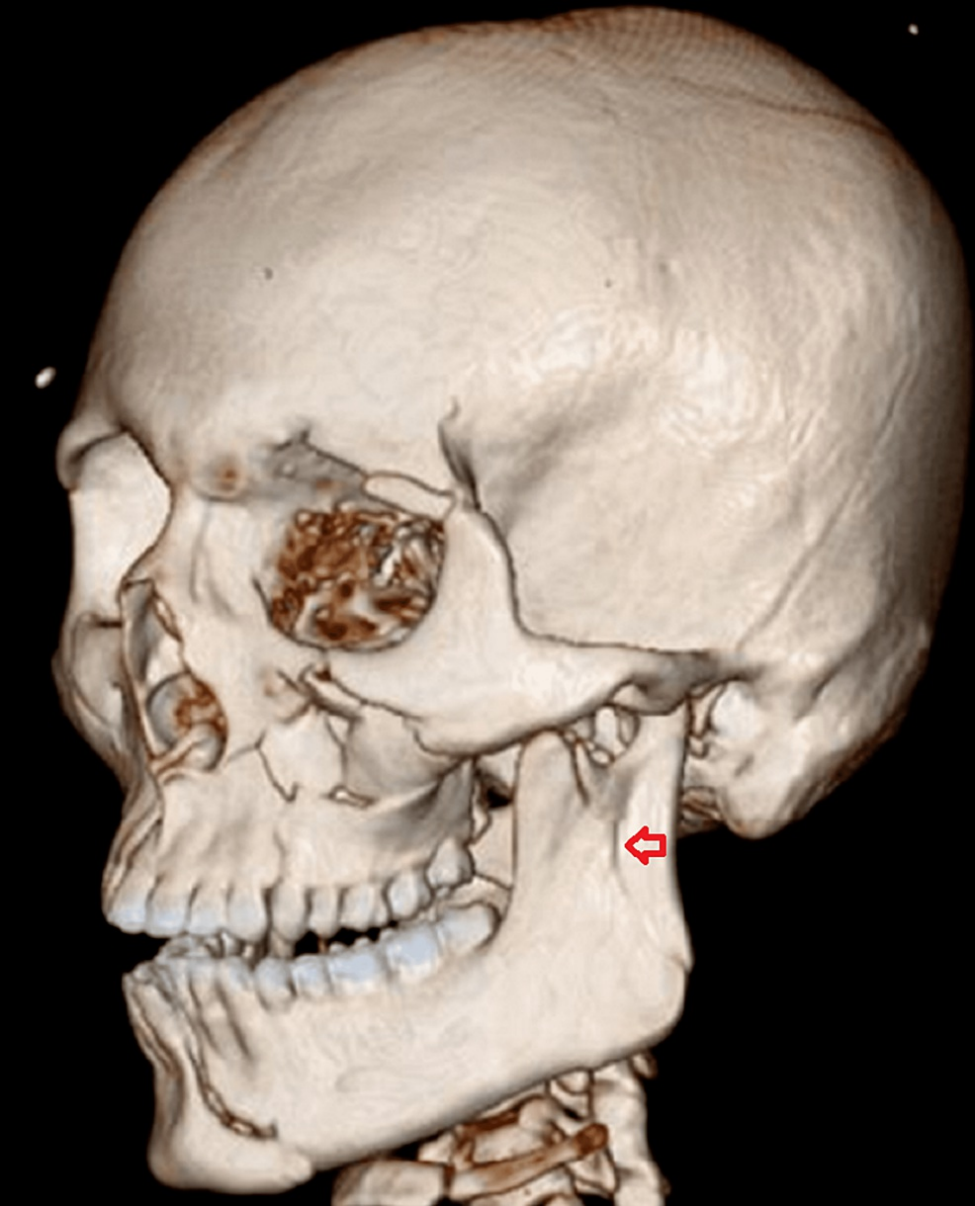
3D reconstructed computed tomographic scan of case II The image shows an oblique fracture line extending from the sigmoid notch to the angle of the mandible on the left side (red arrow).

An orthopantomogram of the patient revealed a left ramus fracture along with a right parasymphysis fracture of the mandible (Figure 12). The fracture line at the left ramus region was oblique from the coronoid of the left side traversing through the ramus up to the posterior border of the mandible 1cm short of the angle of the left side (Figure 13).

**Figure 14:**
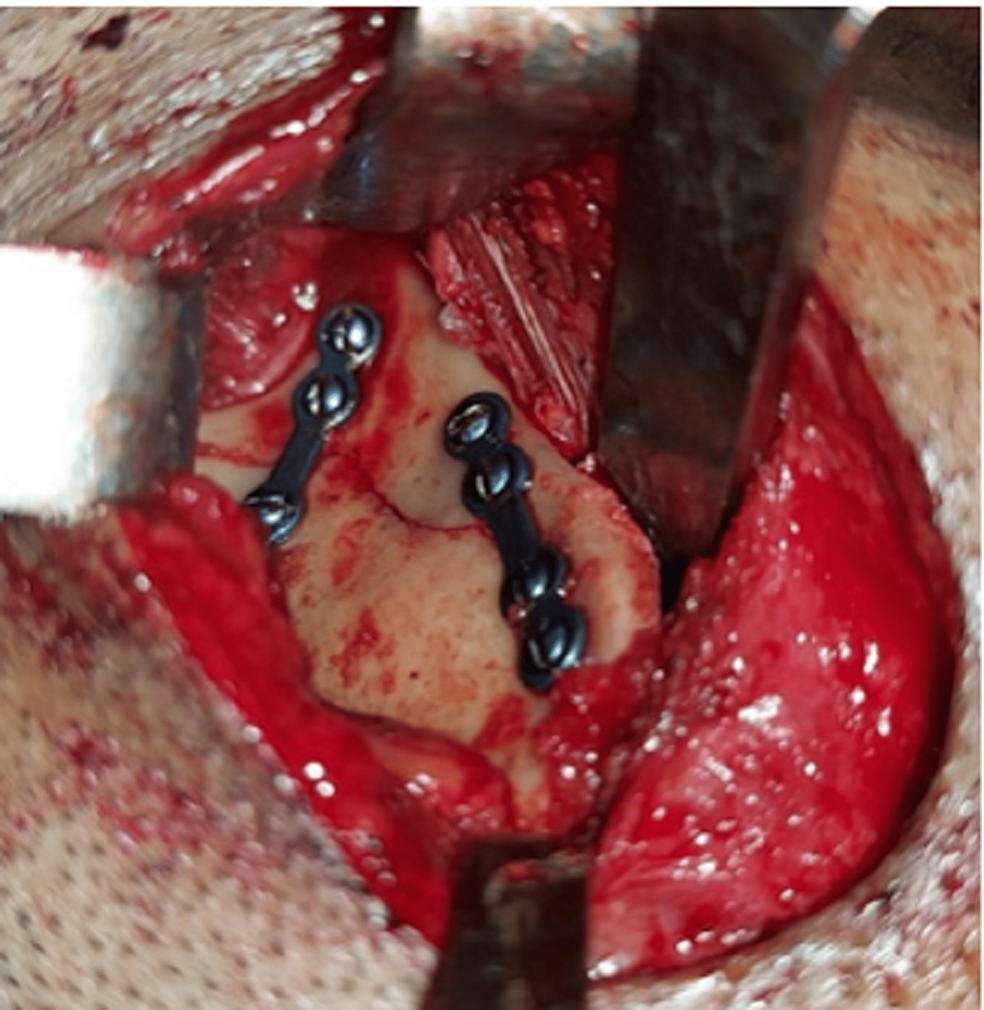
Fixation of the left ramus fracture of case II

**Figure 15:**
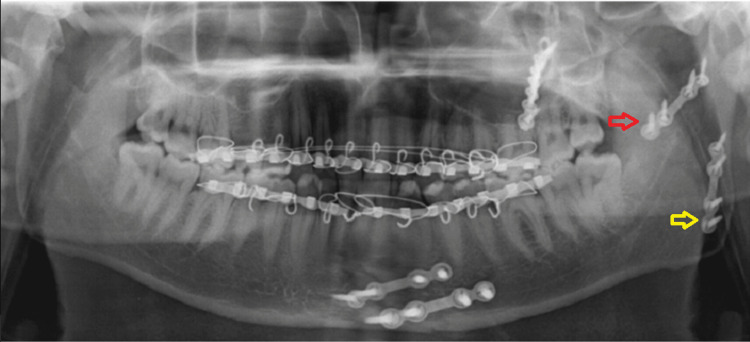
Post-operative OPG of case II OPG: Orthopantomogram The image shows two mini plates one at the superior border of the fracture line above the lingula (red arrow) and the other plate at the posterior border of the mandible (yellow arrow).

The patient was treated surgically with a periangular approach. Fixation was done with two non-compressive mini plates, one at the anterior border of the fracture line below the coronoid and the other plate at the posterior border of the fracture line to avoid the inferior alveolar canal (Figures 14, 15).

Case III

**Figure 16:**
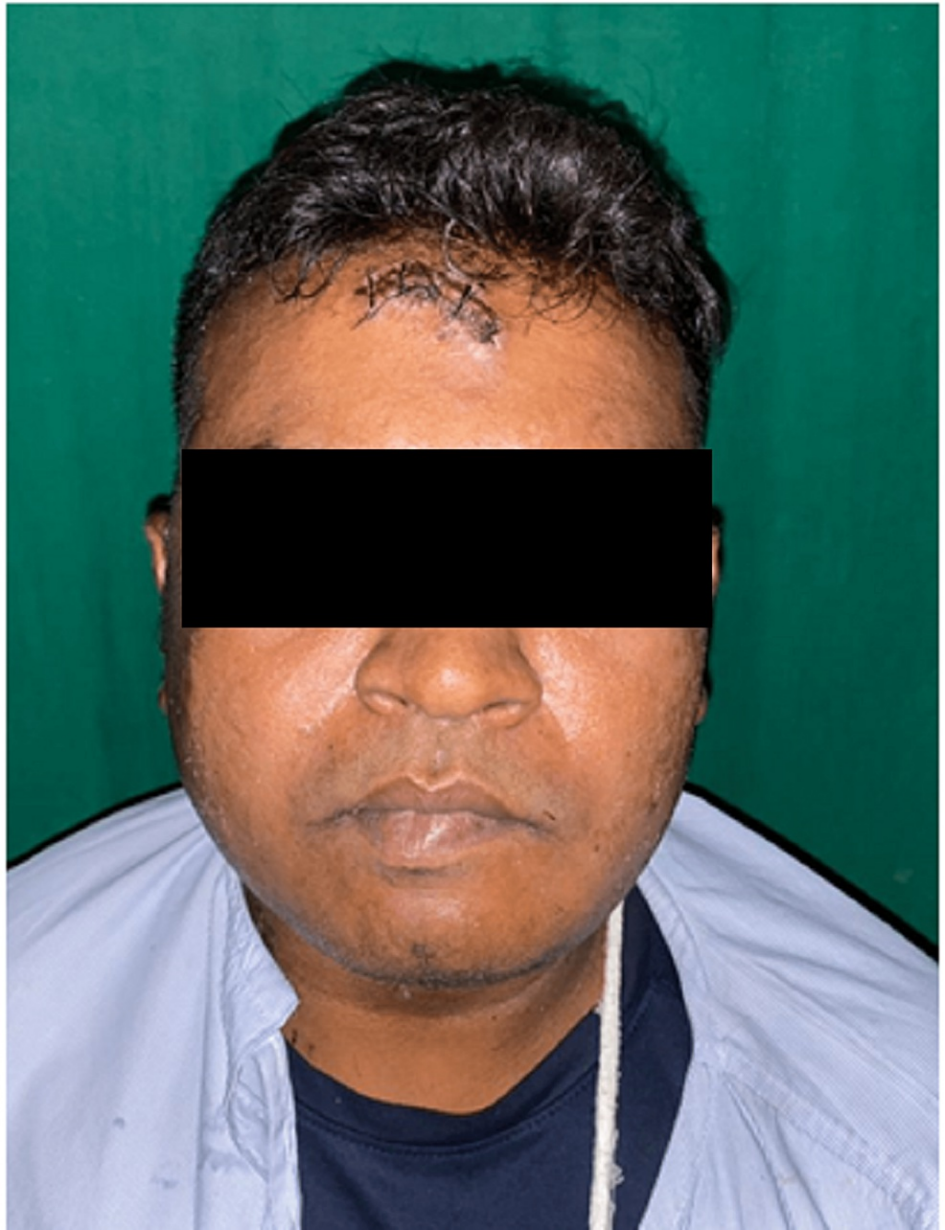
Pre-operative frontal view of case III

A 26-year-old male was brought to the casualty of Acharya Vinoba Bhave Rural Hospital, Wardha, as he was hit by a bullock. The patient had a contused lacerated wound over the left angle region which was sutured. On a detailed clinical examination, step and tenderness were present over the left ramus and right parasymphysis regions. The horizontal and vertical compression tests were positive for tenderness (Figure 16). He had an open bite on the left side and stable occlusion on the right side. There was intersegmental mobility between the mandibular right lateral incisor and canine which was reduced with the help of a stay wire. The patient presented with sublingual haematoma.

**Figure 17:**
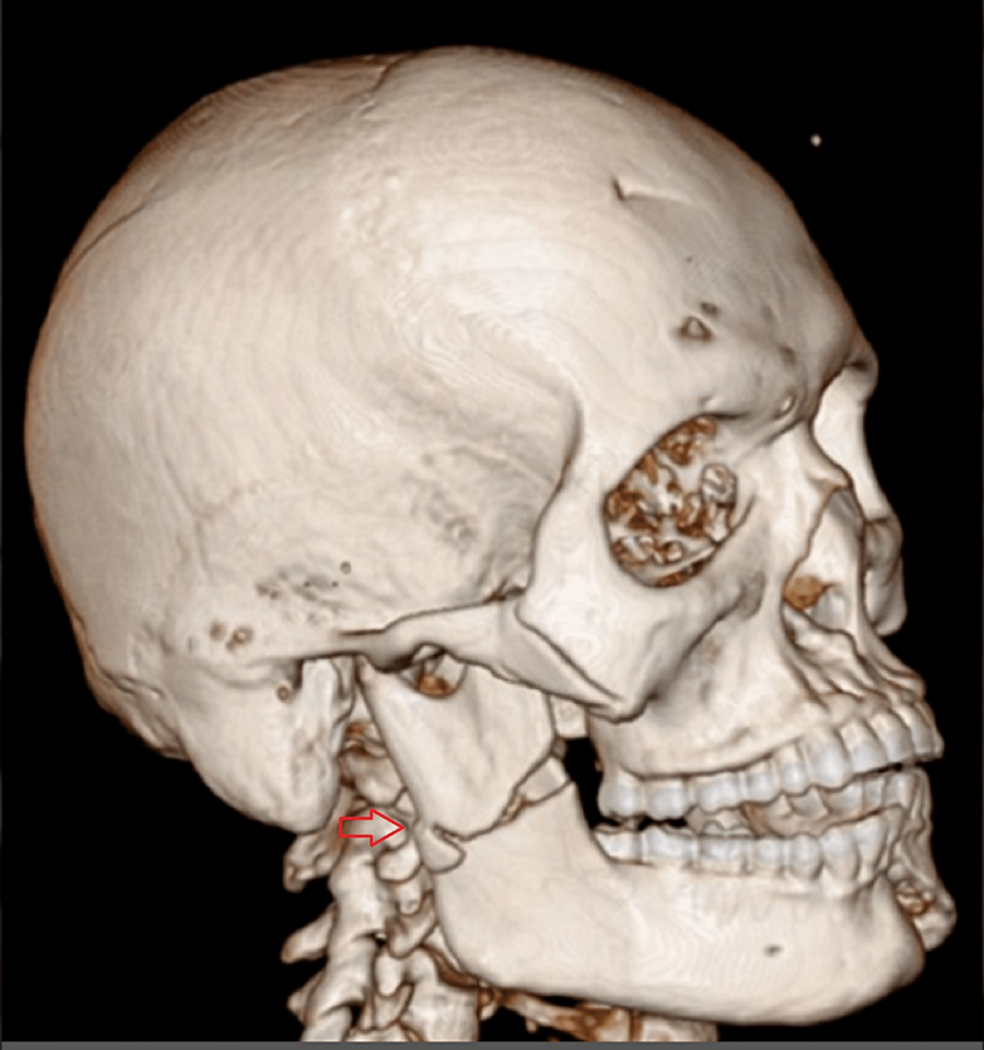
3D reconstructed computer tomographic scan of case III The image shows a horizontal fracture line extending from the anterior border to the posterior border of the ramus on the right side (red arrow).

**Figure 18:**
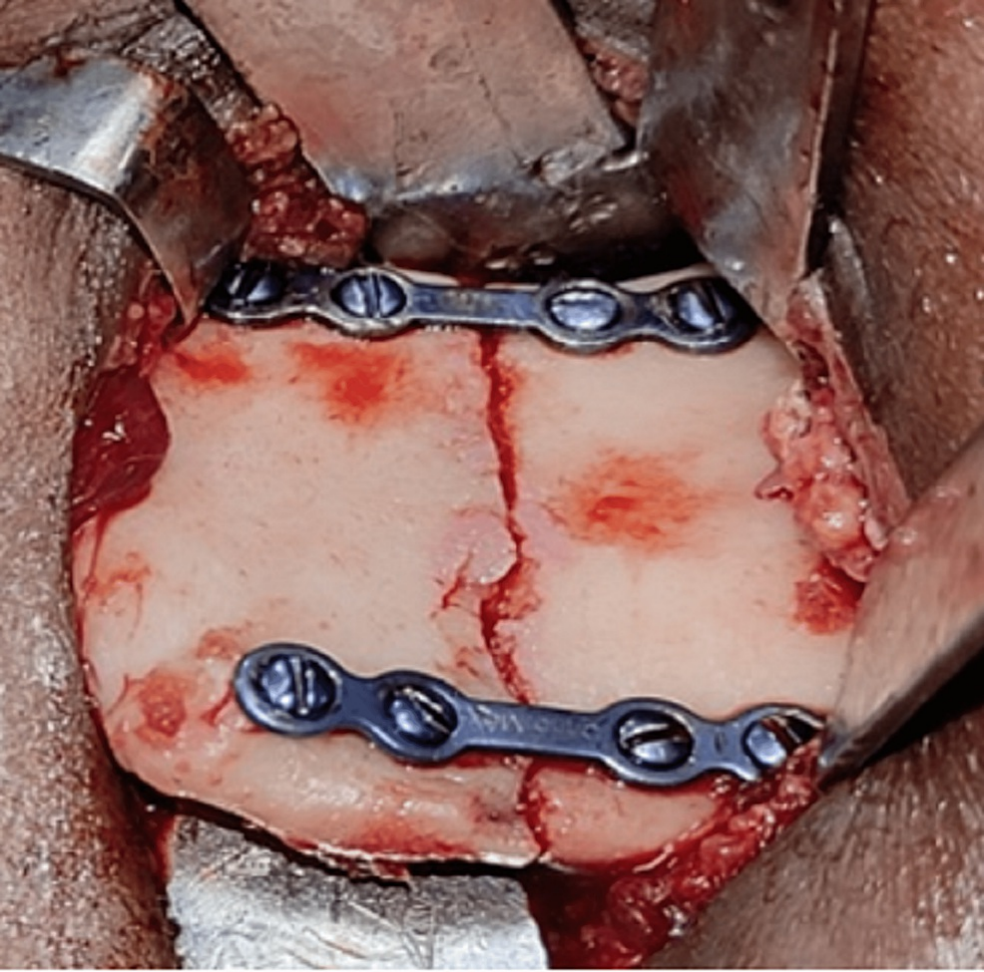
Open reduction and internal fixation of right ramus fracture

**Figure 19:**
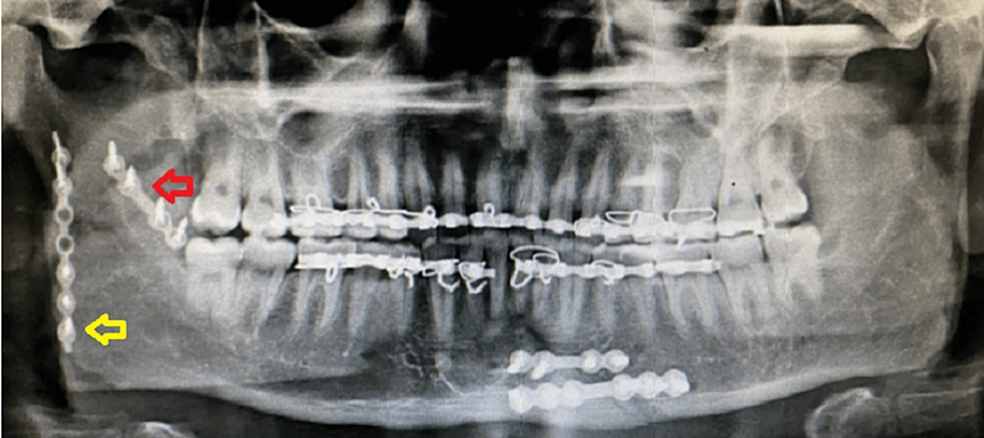
Postoperative orthopantomogram The image shows a mini plate at the external oblique ridge (red arrow) and the other plate at the posterior border of the mandible (yellow arrow) avoiding the mandibular canal

After the clinical examination, the patient underwent a “computed tomographic scan with 3D reconstruction” which showed the presence of a “horizontal fracture line extending from the anterior border and the posterior border of the ramus of the mandible” (Figure 17). After a thorough clinical and radiographical examination, it was found that the patient has left ramus and right parasymphysis fracture. After informed consent, the fractured ramus was treated with open reduction and internal fixation. In which the fractured ramus was reduced and fixed with a periangular incision. The fixation was done with a 2mm 4-hole with gap non-compression miniplate at the anterior border and a 2mm 4-hole with gap non-compression at the posterior border (Figure 18). The patient was given maxillomandibular fixation for four weeks. Post-operatively the patient had stable occlusion bilaterally. The patient was given appropriate antibiotic coverage with analgesics and multivitamins. The surgical site showed satisfactory healing (Figure 19).

Case IV

**Figure 20:**
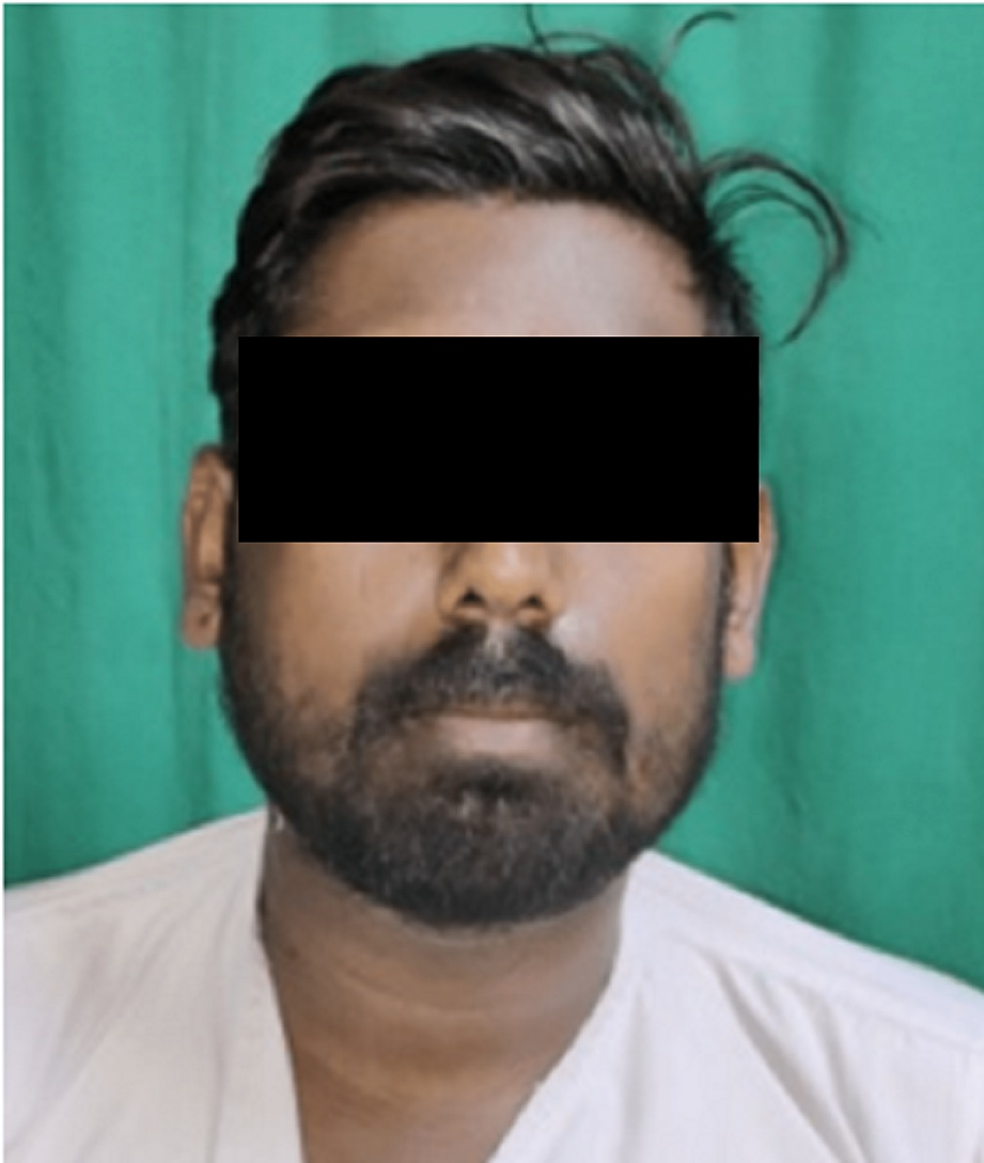
Pre-operative frontal view of case IV

This 23-year-old male patient presented at the casualty with a diffuse swelling over the right preauricular region after RTA due to a fall from a bike (Figure 20). On examination locally, the patient had reduced mouth opening with restricted jaw movements.

**Figure 21:**
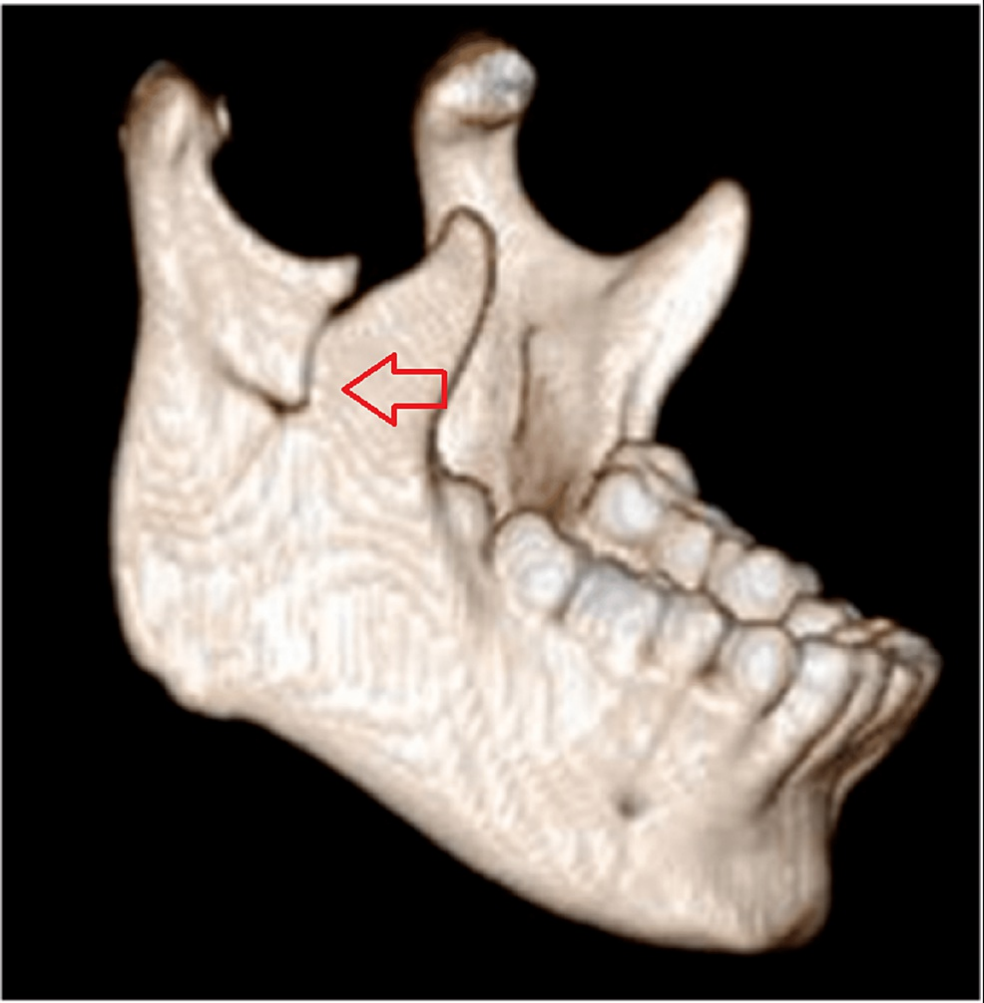
3D computed tomographic scan The image shows the communited fracture of the right mandibular ramus (red arrow).

**Figure 22:**
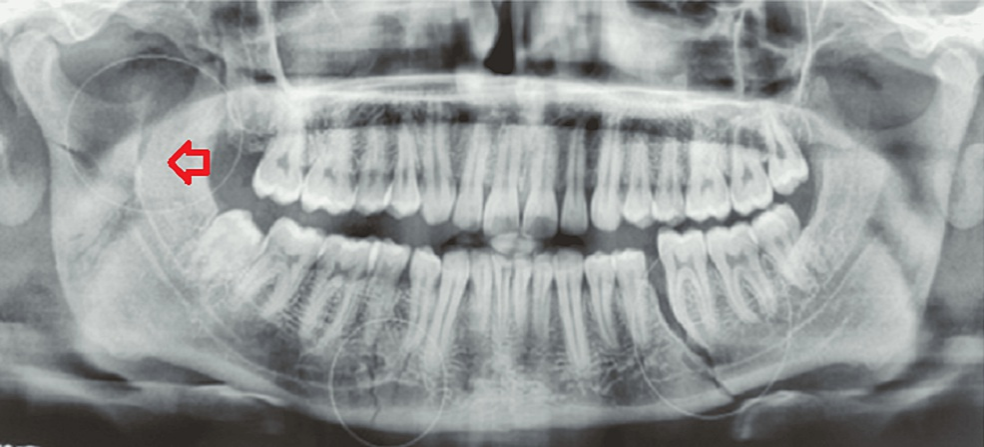
Pre-operative OPG OPG: Orthopantomogram The image shows a communited fracture of the ramus of the right side of the mandible (red arrow).

**Figure 23:**
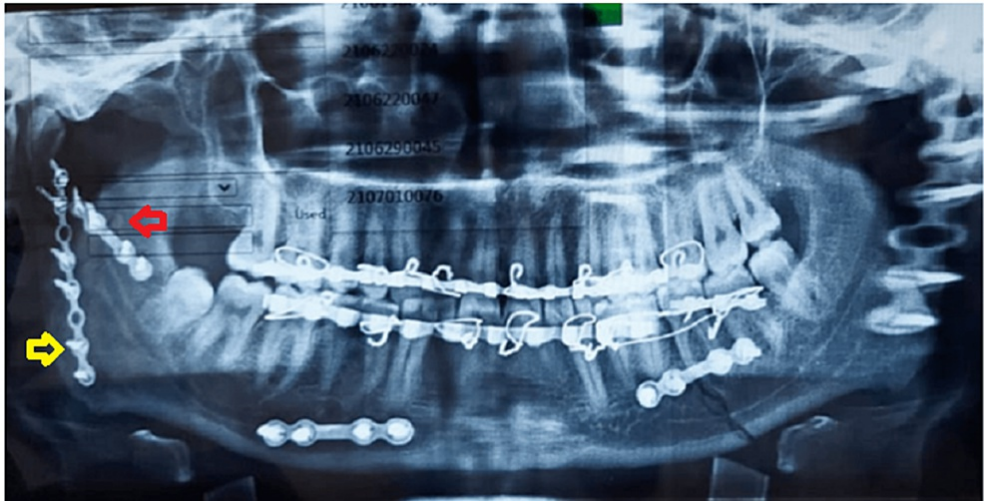
Post-operative OPG OPG: Orthopantomogram The image shows fixation with two mini plates with one plate along the external oblique ridge (red arrow) and the other plate at the posterior border of the mandible (yellow arrow).

There was tenderness present over the left mandibular body and right preauricular region. After subjecting the patient to radiological examination we could elicit that the patient has a left mandibular body and right mandibular ramus fracture. The fracture in the ramus region was communited in type (Figures 21, 22). The fracture was treated by ORIF using a periangular incision. After reducing the fracture anatomically, the fixation was done using an 8-hole continuous non-compression miniplate at the posterior border of the mandible and a 4-hole miniplate with a gap at the external oblique ridge (Figure 23).

## Discussion

Ramus fractures are defined as those in which the fracture line either runs vertically from the sigmoid notch to the posterior border of the mandible or horizontally from the anterior border of the ramus of the mandible to the posterior border of the ramus of the mandible [6]. Ramus fracture is mostly seen in conjunction with other fractures of the face [7]. At our centre, it was found to be accompanied by a parasymphysis fracture of the contralateral side.

ORIF of the ramus is done by various approaches like periangular, transparotid, submasseteric, or intraoral/transbuccal incision. But the transbuccal approach gives inadequate access to the ramus. Open reduction and internal fixation of ramal fractures by two non-compression mini plates provide sufficient anatomical and functional reduction, including length, alignment, and the rotational axis of contiguous fracture fragments, as well as immobilisation, with favourable results and a quick recovery to function [4].

The mandibular ramus fracture as described by Agarwal et al in 2020 is classified into 5 types [8]. In this case series, four of the five types are demonstrated. In case I, the fracture line is extending vertically from the sigmoid notch to the inferior border of the mandible which corresponds to the category of Type I ramus fracture [8]. It is very important to take into consideration the position of the mandibular canal and the lingula. The complications of ORIF of the ramus are paresthesia of the inferior alveolar nerve and hypertrophic scars from the surgery. To prevent that while plating a verticle ramus fracture, two non-compression mini plates are used one superior to the position of the lingual and one at the inferior border of the mandible [9]. Whereas in case II, the fracture line at the left ramus region was oblique from the coronoid of the left side traversing through the ramus up to the posterior border of the mandible 1cm short of the angle of the left side which falls under the category of Type II ramus fracture [10]. In Case III, the fracture line is extending from the anterior border travelling horizontally to the posterior border of the ramus of the mandible corresponding to Type III ramus fracture. In case IV, there is a communited fracture of the ramus which comes under Type V ramus fracture. Type I and Type II are the commonest variants of ramus fracture whereas Type V is the least common variant of ramus fracture. There were satisfactory results seen post-operatively.

## Conclusions

Ramus fracture is an atypical site of the mandible to be fractured owing to its anatomical location. Based on the type of mandibular ramus fracture it becomes imperative to modify the areas of fixation. The use of the non-compression mini-plates confers adequate strength to provide proper anatomical and functional reduction of the fractured ramus of the mandible.

## References

[1] Weihsin H, Thadani S, Agrawal M, Tailor S, Sood R, Langalia A, Patel T (2014). Causes and incidence of maxillofacial injuries in India: 12-year retrospective study of 4437 patients in a tertiary hospital in Gujarat. Br J Oral Maxillofac Surg.

[2] Mijiti A, Ling W, Tuerdi M (2014). Epidemiological analysis of maxillofacial fractures treated at a university hospital, Xinjiang, China: a 5-year retrospective study. J Craniomaxillofac Surg.

[3] Boole JR, Holtel M, Amoroso P, Yore M (2001). 5196 mandible fractures among 4381 active duty army soldiers, 1980 to 1998. Laryngoscope.

[4] Jadhav A, Mundada B, Deshmukh R, Bhutekar U, Kala A, Waghwani K, Mishra A (2015). Mandibular ramus fracture: an overview of rare anatomical subsite. Plast Surg Int.

[5] El-Anwar MW (2018). Changing trends in the treatment of mandibular fracture. Int Arch Otorhinolaryngol.

[6] Kale TP, Kotrashetti SM, Louis A, Lingaraj JB, Sarvesh BU (2013). Mandibular ramus fractures: a rarity. J Contemp Dent Pract.

[7] Koshy JC, Feldman EM, Chike-Obi CJ, Bullocks JM (2010). Pearls of mandibular trauma management. Semin Plast Surg.

[8] Agarwal P, Mehrotra D (2020). Mandibular ramus fractures: a proposed classification. Craniomaxillofac Trauma Reconstr.

[9] Kumari S, Yousuf H, Mishra BP (2022). Mandibular ramus fracture and treatment planning: a review. IJDSIR.

[10] Nardi C, Vignoli C, Pietragalla M (2020). Imaging of mandibular fractures: a pictorial review. Insights Imaging.

